# Influence of traditional processing and genotypes on the antioxidant and antihyperglycaemic activities of yellow-fleshed cassava

**DOI:** 10.3389/fnut.2022.894843

**Published:** 2022-10-14

**Authors:** Babajide Kareem, Emmanuel Anyachukwu Irondi, Emmanuel Oladeji Alamu, Emmanuel Oladipo Ajani, Adebayo Abass, Michael Adesokan, Elizabeth Parkes, Busie Maziya-Dixon

**Affiliations:** ^1^Department of Medical Biochemistry and Pharmacology, Kwara State University, Ilorin, Nigeria; ^2^Food and Nutrition Sciences Laboratory, International Institute of Tropical Agriculture, Lusaka, Zambia; ^3^Food and Nutrition Sciences Laboratory, International Institute of Tropical Agriculture, Lusaka, Zambia; ^4^International Institute of Tropical Agriculture, Dar es Salaam, Tanzania; ^5^International Institute of Tropical Agriculture, Ibadan, Nigeria

**Keywords:** antioxidant activity, bioactive constituents, glycaemic index, starch-digesting enzymes, traditional products, yellow-fleshed cassava

## Abstract

Yellow-fleshed cassava root (YFCR) is processed into traditional products that may influence its bioactivities. In this study, the antioxidant and anti-hyperglycaemic activities of three traditional products (*lafun, fufu* and *gari*) from five genotypes (IITA-TMS-IBA070337, 182961, 182962, 182986, 183044) of YFCR were evaluated. The YFCR genotypes were grown at the International Institute of Tropical Agriculture (IITA) research field, Ibadan. The bioactive constituents (total carotenoids, total phenolics, tannins and total flavonoids), antioxidant [2,2-azinobis (3-ethyl-benzothiazoline-6-sulfonic acid) radical cation (ABTS^•+^) and 1,1-diphenyl-2- picrylhydrazyl radical (DPPH^•^) scavenging capacities, and reducing power], and starch-digesting enzymes (α-amylase and α-glucosidase) inhibitory activities of the products were determined using standard laboratory methods. The glucose response of the products was assessed in human subjects. The concentrations of the bioactive constituents of the products from different genotypes varied significantly (*p* < 0.05). The ABTS^•+^ and DPPH^•^ scavenging capacities and the reducing power of the products also differed significantly (*p* < 0.05), such that the *lafun* from IITA-TMS-IBA182962, IITA-TMS-IBA070337 and IITA-TMS-IBA070337 had the strongest ABTS^•+^ and DPPH^•^ scavenging capacities, and reducing power, respectively. The α-amylase and α-glucosidase inhibitory activities of the three products differed significantly (*p* < 0.05), with the *lafun* from IITA-TMS-IBA070337 and IITA-TMS-IBA07033 having the strongest α-amylase and α-glucosidase inhibitory activity, respectively. Also, the *lafun* from IITA-TMS-182986 had the least glucose response, while the *fufu* from IITA-TMS-IBA070337 had the highest glucose response. Overall, the *lafun* from different genotypes of YFCR had the most potent antioxidant and starch-digesting enzymes inhibitory activities and the least glucose responses. Hence, *lafun* may be a promising dietary intervention targeting oxidative stress, hyperglycaemia, and their resultant type 2 diabetes.

## Introduction

The prevalence of complications associated with hyperglycaemia, such as obesity, type 2 diabetes (T2D), cardiovascular diseases, is significantly increasing worldwide. According to IDF (1), these complications are may become global pandemic by 2030. In Africa, especially in Nigeria, It is speedily becoming a problem in poor economies, where people may still be malnourished (1, 2). Lifestyle and diet have been reported as drivers of hyperglycaemia-associated complications (3). Foods that are rich in carbohydrate contents have been known to increase the postprandial hyperglycaemic level of the blood (4). The consumption of low-fiber and ultra-processed foods are also established contributory risk factors.

Consequently, the association between plant food intake and reduced incidences of complication associated with hyperglycaemia is currently the main focus of some scientific investigations in recent years. This becomes more imperative as the currently available drugs for the treatment and management of some of these complications have been proven to have certain limitations. For example, the clinical use of acarbose for the treatment of T2D is associated with abdominal distention and flatulence in some patients (5).

Provitamin A carotenoids-biofortified (yellow-fleshed) cassava varieties, produced from the biofortification process, serve as a source of energy and important dietary intervention for alleviating vitamin A deficiency, particularly in developing countries (6). Apart from their vitamin A activity, carotenoids and polyphenolics are well-known for their antioxidant properties and protective effects against chronic non-communicable diseases (NCDs) like heart diseases (7), cancer and other complications associated with hyperglycaemia (8, 9). In developing countries, cassava is traditionally processed into different products, including gari, fufu, boiled and unfermented flour (10). As the report of Maziya-Dixon et al. (11) indicated, processing methods reduce the levels of carotenoids in provitamin A-biofortified cassava varieties. Thus, identifying the yellow-fleshed cassava roots (YFCR) genotype(s) and the traditional products with potent antioxidant and starch-digesting enzymes inhibitory activities, and a low glucose response could make biofortified cassava a cost-effective dietary intervention for postprandial hyperglycaemia and oxidative stress.

Thus, the purpose of this study was to determine the effect of traditional processing on the antioxidant, starch-digesting enzymes inhibitory activities and glucose response of YFCR genotypes.

## Materials and methods

### Materials

Samples of five YFCR genotypes (IITA-TMS-IBA182962, 183044, 182961 and 182986), were collected from the International Institute of Tropical Agriculture's (IITA) research farm in Ibadan, Nigeria, from the institute's cassava breeding unit. Each genotype's roots were thoroughly washed with water to remove all dirt and adherent sand particles, and then divided into three 5 kg portions. Each portion was then processed into *fufu, lafun, and gari*.

### Processing of yellow-fleshed cassava genotypes into *fufu, lafun* and *gari*

**Figure 1:**
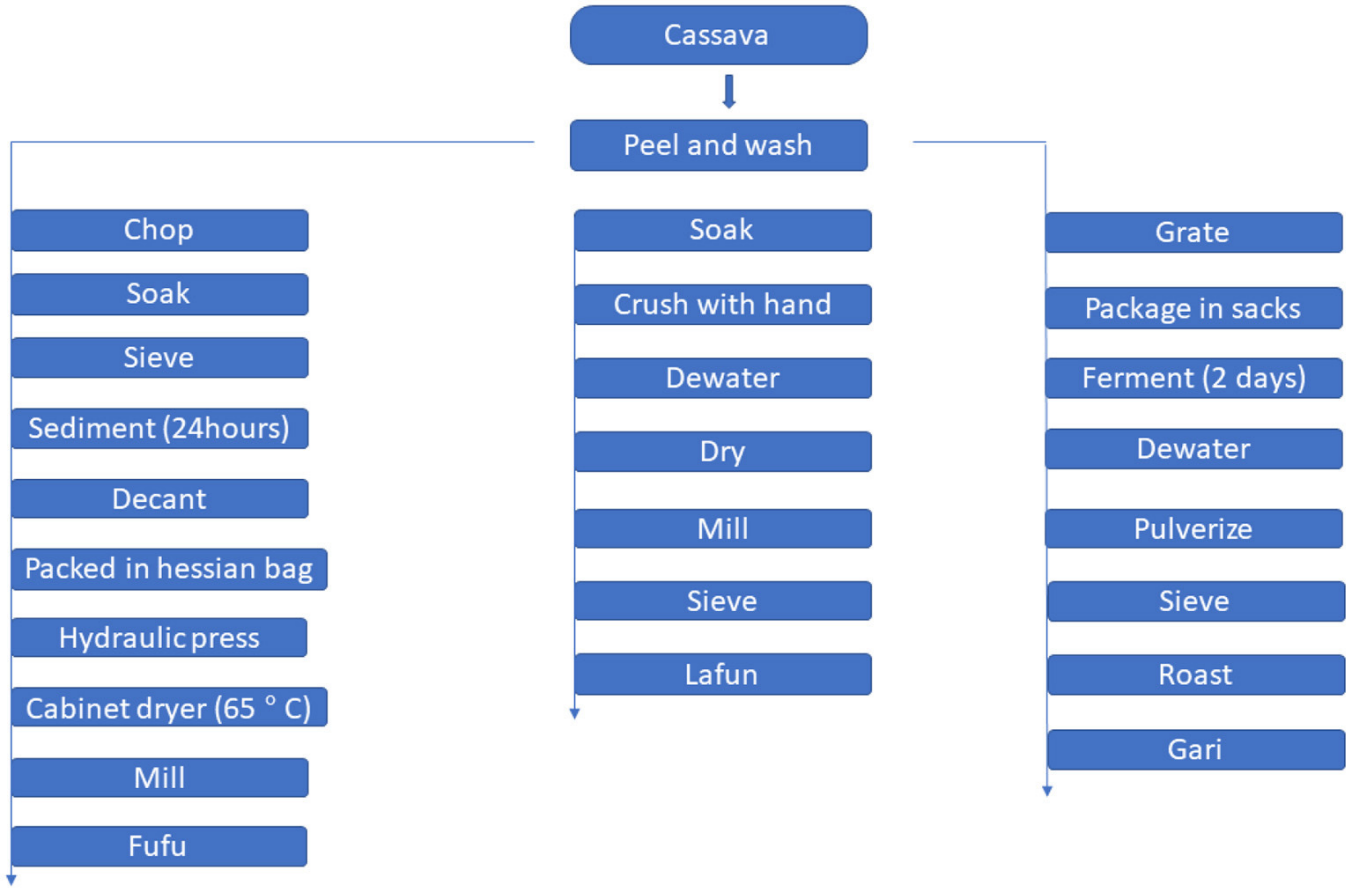
A flow chart for traditional processing of cassava to *gari, fufu* and *lafun*.

A flow chart for traditional processing of cassava to *gari, fufu* and *lafun* is shown in Figure 1.

#### Processing of fufu

The traditional processing method described by Sanni et al. (12) was modified slightly to produce *fufu*. YFCR that were freshly harvested were peeled and thoroughly washed with tap water to remove any adhered sand. Approximately 5 kg of peeled YFCR was cut into ~15 cm-long chunks with a stainless-steel hand knife and steeped in 5 liters of water in a plastic bowl for 3–5 days at ambient room temperature (28–32°C) for retting. The retted roots were manually broken and the fibers were sieved out. The mash was manually sieved using a mesh cloth sieve. In a large plastic bowl, sieved mash samples were allowed to sediment for 24 h. Following sedimentation, the water was decanted and the sediment was washed with additional water. The sediment (*fufu* mash) was dewatered by placing it in Hessian sacks and pressing it with a hydraulic press to remove excess moisture. The pressed mash was then dried for 8 h at 65°C in a cabinet dryer, before being milled in a stainless-steel hammer mill.

#### Processing of lafun

*Lafun* was processed according to Cassbiz (13) method, with a few modifications. YFCR was peeled, washed, and soaked in a bowl for 4 days before being crushed by hand and dewatered using a hydraulic press. The crushed product was dried at room temperature for 3–4 days before being milled and sieved to remove fiber. The resulting powder was cooked in boiling water for 5 min to form a stiff paste.

#### Processing of gari

The YFCR was processed into *gari* according to the procedure described by Abass et al. (14). The roots were washed, peeled, and grated into mash as soon as they were harvested. The mash was packed into sacks and left to ferment for 2 days in a fermentation trough. The fermented mash was dewatered by passing it through a hydraulic press. The fibrous materials were pulverized and sieved to separate them from the dewatered cakes. The flour was roasted at 125°C for 25 min, stirring constantly, until the granules became creamy and free-flowing.

### Preparation of methanolic extract of *fufu, lafun* and *gari*

Methanolic extract of each of the three products was prepared according to Chan et al. (15) method with a little modification. Methanol (25 mL) was added to 0.5 g of the sample in a covered centrifuge tube (50 mL) for 12 h. The mixture was shaken for 1 h using a mechanical shaker, after which it was spinned in a centrifuge at 3,000 g for 10 min. Then the supernatant (methanolic extract) was stored at −4°C until analysis.

### Determination of bioactive constituents of *lafun, fufu and gari*

#### Total carotenoids concentration determination

The total carotenoid concentration in *lafun, fufu and gari* was quantified by adopting the protocol reported by Maziya-Dixon et al. (11). The carotenoid analysis was performed under dim light, and the extracts were collected into an aluminum foil-wrapped vial to prevent the degradation of the carotenoids.

#### Tannins concentration determination

The concentration of tannin in the products was determined using the method described by Joslyn (16), with a little modification. A portion of the sample (0.5 g) was dissolved in 5 mL of 1% HCl in methanol for 15 min. The mixture was vortex and centrifuged at 3,000 g for 10 min, following which 0.1 mL of the supernatant was addedto 7.5 mL of distilled water in a test tube. Next, 0.5 mL of Folin-Dennis reagent and 1 mL of Na_2_CO_3_ solution were added to the mixture, and the volume was made up to 10 mL with 0.9 mL of distilled water. The mixture was incubated for a period of 30 min at room temperature, and the absorbance was read at 760 nm. The tannins concentration of the sample, presented as tannic acid equivalent (TAE) in m/g was calculated from a tannin calibration curve.

### Total phenolics concentration determination

The Folin–Ciocalteu method reported by Chan et al. (15) was employed to assay the total phenolic concentration of the methanolic extract of each product. A 300 μL portion of each product's methanolic extract was dispensed into a test tube. Next, 1.5 mL of Folin–Ciocalteu reagent (diluted 10 times with distilled water) and 1.2 mL of Na_2_CO_3_ solution (7.5% w/v) was added. The reaction mixture was shaken and incubated at room temperature for a period of 30 min before the absorbance (at 765 nm) was measured against a blank prepared by dispensing 300 μL of distilled water in place of the sample extract. The total phenolic concentration was presented in mg/g as gallic acid equivalents (GAE).

#### Total flavonoids concentration determination

The protocol described by Asha et al. (17) was used to determine the total flavonoids content of the products' methanolic extracts. The extract (0.5 mL) was mixed with methanol (1.5 mL), 10% aluminum chloride (0.1 mL), 1M potassium acetate (0.1 mL) and of distilled water (2.8 mL) were added. The mixture was incubated for a period of 30 min at room temperature, before the absorbance was read at 514 nm. The total flavonoids content of the extract was expressed as quercetin equivalent (QE) in mg/g.

#### 2,2-azinobis (3-ethyl-benzothiazoline-6-sulfonic acid) radical cation (ABTS^•+^) scavenging assay

The ABTS^•+^ scavenging capacity of the products' extracts was carried out following the method reported by Re et al. (18). To prepare the working solution of ABTS^•+^ reagent, aqueous solutions of 7 millimoles/L ABTS^•+^ and 2.45 millimoles/L K_2_S_2_O_8_ were mixed in the same proportion (v/v) and incubated for 16 h at room temperature in a dark enclosure. After that, the absorbance reading of the ABTS^•+^ working reagent at 734 nm was adjusted to 0.70 ± 0.02 by diluting with ethanol (95%). A portion of of the ABTS^•+^ reagent (2.0 mL) and the extract (0.2 mL) was dispensed in a test tube, mixed well and incubated in a dark enclosure for a period of 15 min at room temperature, after which the absorbance reading was taken at 734 nm. ABTS^•+^ scavenging capacity of each extract, presented in trolox equivalent antioxidant capacity (TEAC), was calculated using a trolox calibration curve.

#### Assay for 2, 2-diphenyl-2-picrylhydrazyl radical (DPPH^•^) scavenging capacity

The DPPH^•^ scavenging capacity of the products' extract was conducted by adopting the procedure described by Cervato et al. (19). Vitamin C was used as a positive control in this assay. A mixture of different concentrations (totaling 1 mL) of the extract (or ascorbic acid) and DPPH^•^ solution (3.0 mL, 60 μM) was incubated in a dark enclosure at room temperature for a period of 30 min. Thereafter, the absorbance reading at 517 nm was taken and the DPPH^•^ scavenging capacity of the extract, presented as SC_50_ (extract concentration that scavenged 50% of DPPH^•^).

#### Ferric [iron (III)] reducing power assay

The protocol reported by Oyaizu (20) was adopted to determine the ability of the products' extracts to reduce FeCl_3_ solution. The sample extract (2.5 mL) was mixed with 2.5 mL of 200 mM sodium phosphate buffer (pH 6.6), and 2.5 mL of 1% potassium ferricyanide. The mixture was incubated at 50°C for a period of 20 min, following which 2.5 mL of 10% trichloroacetic acid was added. The mixture was spinned at 650 × g for a period of 10 min, and the supernatant was divided into 2.5 mL portions in different test tubes. Next, distilled water (2.5 mL) and 0.1% ferric chloride solution (1 mL) were added to the content of each test tube, and the absorbance reading at 700 nm was taken. The reducing power of the extracts, presented as gallic acid equivalent in mg per gram, was calculated using a gallic acid calibration curve.

### Determination of digestive enzymes (α-amylase and α-glucosidase) inhibitory activity, *in vitro*

#### Alpha-amylase inhibition assay

The inhibition of alpha-amylase by the products' methanolic extract was determined using the method described by Kwon et al. (21). In this assay, porcine pancreas -amylase (EC 3.2.1.1) and soluble starch (substrate) purchased from sigma, st louis, USA were used. A mixture of 500 μL of different dilutions of the product's extract and 500 μL of 0.02 M sodium phosphate buffer (pH 6.9 with 0.006 M NaCl) containing 0.5 mg/mL α-amylase solution was subjected to incubation at 37°C for a period of 10 min. After that, 1% starch solution in 0.02 M sodium phosphate buffer (500 μL) was added. The reaction mixture was subjected to incubation at 37°C for a period of 15 min, followed by 1.0 mL of DNSA color reagent (1% 3, 5-dinitrosalicylic acid and 12% sodium potassium tartrate in 0.4 M NaOH) to terminate the reaction. Next, the reaction mixture was incubated for 5 min in a boiling water bath, and diluted with 10 mL of distilled water, after which it was left to cool to room temperature. The absorbance was read at 540 nm, and the percentage α-amylase inhibition was calculated using the following equation:


(1) $$\begin{array}{r} \%\;Inhibition\;=\;\left[\left(AR\;-\;AS\right)/AR\right]\;\times \;100 \end{array}$$

*Where AR is the mean absorbance reading of the reference; AS is the mean absorbance reading of the sample*.

### Alpha-glucosidase inhibition assay

The protocol described by Kim et al. (22) was adopted to determine the capacity of the products' extract to inhibit the starch hydrolytic activity of α-glucosidase. In this assay, 50 μL of α-glucosidase (5 units) was incubated with 50 μL of different concentrations of each product's extract at 37°C for a period of 15 min. Next, 3 mM of the substrate (para-nitrophenylglucopyranoside, PNPG) dissolved in 20 mM phosphate buffer, pH 6.9, was added to initiate the α-glucosidase-catalyzed hydrolytic reaction. The hydrolysis of PNPG occurred for a period of 20 min at 37°C, following which it was terminated by adding 0.1 M Na_2_CO_3_ (2 mL). The absorbance of the yellow *p*-nitrophenol released from the hydrolysis of PNPG was read at 400 nm. The percentage α-glucosidase inhibition was calculated using the following equation:


(2) $$\begin{array}{r} \%\;Inhibition\;=\;\left[\left(AR\;-\;AS\right)/AR\right]\;\times \;100 \end{array}$$

*Where AR is the mean absorbance reading of the reference; AS is the mean absorbance reading of the sample*.

### Glucose response experiment

Ethical clearance (MOH/KS/EU/777/497) to conduct the glucose response experiment was obtained from Kwara State Ministry of Health, Ilorin, Nigeria. Nineteen apparently healthy, non-diabetic and non-obese subjects, comprising 8 male and 11 female students of Kwara State University, Malete, Ilorin, Nigeria, aged 18–25 years, volunteered to participate in the study. Informed consent was obtained from the volunteers.

All the volunteers were first subjected to an oral glucose challenge after overnight fasting by drinking 50 g of glucose in 25 mL of potable water. Their blood glucose level was measured at 0, 30, 60, and 90 min using a glucometer (Accu-Chek Active). After that, they were randomly distributed into groups of three volunteers per sample (*fufu, lafun* and *gari*). Next, 50 g of reconstituted *fufu*/*lafun*/*gari* was served (on different days) with an equal portion of *ewedu* soup and fish. The formula and mode of cooking the soup were consistent throughout the study.

### Data analysis

Data of replicate experiments were subjected to one-way ANOVA, followed by Duncan multiple range test at 95% confidence interval, using SPSS statistical software (version 17). In addition, GraphPad Prism 5 for Windows, GraphPad Software, San Diego, California USA, www.graphpad.com was used to plot the histograms.

## Results

### Bioactive constituents of *fufu, lafun and gari* processed from five YFCR genotypes

The bioactive constituents (total carotenoids, total phenolics, tannins and total flavonoids contents) of the *fufu, lafun and gari* processed from the five YFCR genotypes (IITA-TMS-IBA182962, 183044, 182961 and 182986) are presented in Table 1. Overall, the total carotenoid level ranged from 0.38 to 10.16 μg/g in *gari* from IITA-TMS-IBA070337 and *fufu* from IITA-TMS-IBA182986, respectively. Further, the total carotenoid content of *fufu* from IITA-TMS-IBA182986 was significantly (*p* < 0.05) higher than those of *fufu, lafun* and *gari* from the other four genotypes (IITA-TMS-IBA070337, IITA-TMS-IBA182961, IITA-TMS-IBA182962 and IITA-TMS-IBA183044). The total phenolics level ranged from 0.1 to 0.38 GAE mg/g in *lafun* from IITA-TMS-IBA182986 and *gari* from IITA-TMS-IBA182962, respectively. Tannins content was in the range of 0.003–0.019 TAE mg/g in *fufu* from IITA-TMS-IBA070337 and *lafun* from IITA-TMS-IBA182962, while the total flavonoids ranged from 0.03 to 0.24 QE mg/g in *fufu* and *lafun* from IITA-TMS-IBA070337 and *gari* from IITA-TMS-IBA182961, respectively. Generally, the levels of the bioactive constituents of the products varied significantly (*p* < 0.05) across the genotypes.

**Table 1:** Bioactive constituents of *fufu, lafun and gari* from five provitamin A biofortified yellow-fleshed cassava genotypes.

**Accession No**.	**Product**	**Total carotenoids (μg/g)**	**Total phenolics (mg GAE/g)**	**Tannins (mg TAE/g)**	**Total flavonoids (mg QE/g)**
IITA-TMS-IBA070337	*Fufu*	0.79 ± 0.00^a^	0.21 ± 0.01^b^	0.00 ± 0.00^a^	0.03 ± 0.00^a^
	*Gari*	0.38 ± 0.02^a^	0.22 ± 0.03^b^	0.01 ± 0.00^c, d^	0.04 ± 0.01^a^
	*Lafun*	0.88 ± 0.00^a^	0.24 ± 0.02^b, c, d^	0.01 ± 0.00^d, e^	0.03 ± 0.00^a^
IITA-TMS-IBA182961	*Fufu*	7.30 ± 0.01^g^	0.27 ± 0.04^b, c, d^	0.01 ± 0.00^a, b^	0.07 ± 0.00^b^
	*Gari*	6.09 ± 0.00^f^	0.31 ± 0.07^e^	0.01 ± 0.00^h, i^	0.24 ± 0.06^g^
	*lafun*	9.29 ± 0.01^h^	0.24 ± 0.01^b, c, d^	0.01 ± 0.00^h, i^	0.13 ± 0.01^d, e^
IITA-TMS-IBA182962	*Fufu*	2.26 ± 0.02^b^	0.29 ± 0.06^c, d, e^	0.01 ± 0.00^b, c^	0.10 ± 0.02^b, c, d^
	*Gari*	9.31 ± 0.01^h^	0.37 ± 0.07^f^	0.02 ± 0.00^i^	0.18 ± 0.02^f^
	*Lafun*	6.43 ± 0.01^f^	0.30 ± 0.01^d, e^	0.02 ± 0.00^j^	0.15 ± 0.01^e, f^
IITA-TMS-IBA182986	*Fufu*	10.16 ± 1.20^i^	0.33 ± 0.02^e, f^	0.01 ± 0.00^c^	0.09 ± 0.01^b, c^
	*Gari*	3.75 ± 0.50^c^	0.38 ± 0.01^f^	0.02 ± 0.00^j^	0.11 ± 0.02^c, d^
	*Lafun*	4.71 ± 0.02^d^	0.10 ± 0.00^a^	0.01 ± 0.00^g, h^	0.08 ± 0.00^b^
IITA-TMS-IBA183044	*Fufu*	6.43 ± 0.08^f^	0.24 ± 0.03^b, c, d^	0.01 ± 0.00^c^	0.07 ± 0.02^b^
	*Gari*	5.22 ± 0.02^e^	0.30 ± 0.07^d, e^	0.01 ± 0.00^e, f^	0.16 ± 0.01^f^
	*Lafun*	5.45 ± 0.00^e^	0.23 ± 0.01^b, c^	0.01 ± 0.00^f, g^	0.12 ± 0.00^d, e^

*Data are the results of replicate determinations. Values having different superscript alphabets along the same column are significantly different at p < 0.05. GAE, gallic acid equivalent; TAE, tannic acid equivalent; QE, quercetin equivalent*.

### Antioxidant activity of *fufu, lafun and gari* processed from five YFCR genotypes

The *in vitro* antioxidant activity of *fufu, lafun and gari* processed from five YFCR genotypes are presented in Table 2. The ABTS^•+^ scavenging ability ranged from 5.96 to 8.82 TEAC mmol/g in *fufu* from IITA-TMS-IBA182961 and *lafun* from IITA-TMS-IBA182962, respectively. There was a significant difference (*p* < 0.05) in the ABTS^•+^ scavenging ability of the three products across the genotypes. The reducing power also varied significantly (*p* < 0.05) among the products from the different genotypes and ranged from 0.34 to 0.97 GAE mg/g in *fufu* from IITA-TMS-IBA070337 and *lafun* from IITA-TMS-IBA182962, respectively. The DPPH^•^ scavenging ability, expressed as the products' extract concentration that scavenged DPPH^•^ by 50% (SC_50_) also varied significantly (*p* < 0.05) among products from different genotypes and ranged from 85.61 to 398.30 mg/mL in *lafun* from IITA-TMS-IBA182961 and *fufu* from IITA-TMS-IBA07033, respectively.

**Table 2:** Antioxidant activity of *fufu, lafun and gari* from five provitamin A biofortified yellow-fleshed cassava genotypes.

**Accession No**.	**Product**	**SC_50_ (mg/mL)**	**ABTS^•+^ scavenging ability (TEAC mmol/g)**	**Reducing power (GAE mg/g)**
IITA-TMS-IBA070337	*Fufu*	398.31 ± 52.08^i^	6.78 ± 0.78^a, b^	0.34 ± 0.01^a^
	*gari*	99.47 ± 12.59^a, b^	6.67 ± 0.55^a, b^	0.34 ± 0.02^a^
	*lafun*	177.46 ± 42.68^g^	8.39 ± 0.89^c, d^	0.39 ± 0.01^a^
IITA-TMS-IBA182961	*fufu*	282.72 ± 24.18^h^	5.95 ± 0.41^a^	0.63 ± 0.01^d^
	*gari*	113.95 ± 3.53^a, b, c, d^	7.02 ± 0.18^a, b^	0.50 ± 0.01^b, c^
	*lafun*	85.61 ± 4.71^a^	8.78 ± 0.25^d^	0.91 ± 0.03^f^
IITA-TMS-IBA182962	*fufu*	181.11 ± 26.62^g^	7.27 ± 1.13^b, c^	0.78 ± 0.12^e^
	*gari*	147.69 ± 16.64^d, e, f, g^	7.28 ± 0.45^b, c^	0.53 ± 0.01^c^
	*lafun*	95.31 ± 10.81^a, b^	8.81 ± 0.44^d^	0.97 ± 0.06^f^
IITA-TMS-IBA182986	*fufu*	173.85 ± 27.21^f, g^	7.39 ± 0.73^b, c^	0.77 ± 0.04^e^
	*gari*	123.22 ± 9.39^b, c, d, e^	7.28 ± 0.68^b, c^	0.59 ± 0.06^d^
	*lafun*	108.18 ± 7.24^a, b, c^	7.74 ± 0.93^b, c, d^	0.73 ± 0.04^e^
IITA-TMS-IBA183044	*fufu*	153.51 ± 18.52^e, f, g^	7.32 ± 1.07^b, c^	0.65 ± 0.01^d^
	*gari*	129.71 ± 1.99^b, c, d, e^	7.00 ± 0.64^a, b^	0.45 ± 0.01^b^
	*lafun*	138.62 ± 23.53^c, d, e, f^	7.20 ± 0.59^b^	0.78 ± 0.04^e^

*Data are the results of replicate determinations. Values having different superscript alphabets along the same column are significantly different at p < 0.05. SC_50_: concentration of extract that scavenged 50% of DPPH^•^; TEAC, trolox equivalent antioxidant capacity; GAE, gallic acid equivalent*.

### Starch-digesting enzymes (α-amylase and α-glucosidase) inhibitory activity of *fufu, lafun and gari* processed from five YFCR genotypes

The α-amylase and α-glucosidase inhibitory activity of extracts of the *fufu, lafun and gari* from the five genotypes of YFCR are shown in Table 3. The α-amylase IC_50_ differed significantly (*p* < 0.05) among the three products from the different genotypes and ranged from 24.57 to 83.88 mg/mL in *lafun* from IITA-TMS-IBA070337 to *lafun* from IITA-TMS-IBA182962, respectively. On the other hand, the α-glucosidase IC_50_ ranged from 32.36 to 62.56 mg/mL in *lafun from* IITA-TMS-IBA07033 and *lafun* from IITA-TMS-IBA182961, respectively.

**Table 3:** Starch-digesting enzymes inhibitory activity of extracts of *fufu, lafun and gari* from five provitamin A biofortified yellow-fleshed cassava genotypes.

**Accesion No**.	**Products**	**Alpha-amylase IC_50_ (mg/mL)**	**Alpha-glucosidase IC_50_ (mg/mL)**
IITA-TMS-IBA070337	*Fufu*	32.24 ± 2.30^a, b, c^	36.97 ± 4.53^a^
	*Gari*	28.38 ± 1.80^a, b^	35.83 ± 3.68^a^
	*Lafun*	24.57 ± 1.20^a^	32.36 ± 3.82^a^
IITA-TMS-IBA182961	*Fufu*	39.94 ± 2.10^c, d, e^	50.83 ± 6.51^b^
	*Gari*	28.72 ± 1.90^a, b^	40.98 ± 4.53^a, b^
	*lafun*	24.83 ± 0.23^a^	62.56 ± 6.65^c^
IITA-TMS-IBA182962	*fufu*	40.20 ± 2.50^c, d, e^	38.22 ± 4.95^a^
	*gari*	60.13 ± 3.60^f^	37.10 ± 4.37^a^
	*lafun*	83.88 ± 3.80^g^	43.73 ± 5.37^a, b^
IITA-TMS-IBA182986	*fufu*	35.19 ± 2.50^b, c, d^	39.31 ± 2.97^a^
	*gari*	44.99 ± 2.60^e^	34.60 ± 3.96^a^
	*lafun*	38.46 ± 2.20^c, d, e^	39.19 ± 4.53^a^
IITA-TMS-IBA183044	*fufu*	39.80 ± 2.30^c, d, e^	35.63 ± 3.96^a^
	*gari*	40.44 ± 3.10^d, e^	39.62 ± 4.38^a^
	*lafun*	34.43 ± 1.30^b, c, d^	36.23 ± 4.53^a^

*Data are the results of replicate determinations. Values having different superscript alphabets along the same column are significantly different at p < 0.05. IC_50_: concentration of extract that inhibited the enzyme activity by 50%*.

### Blood glucose response of *fufu, lafun* and *gari* processed from five YFCR genotypes

**Figure 2:**
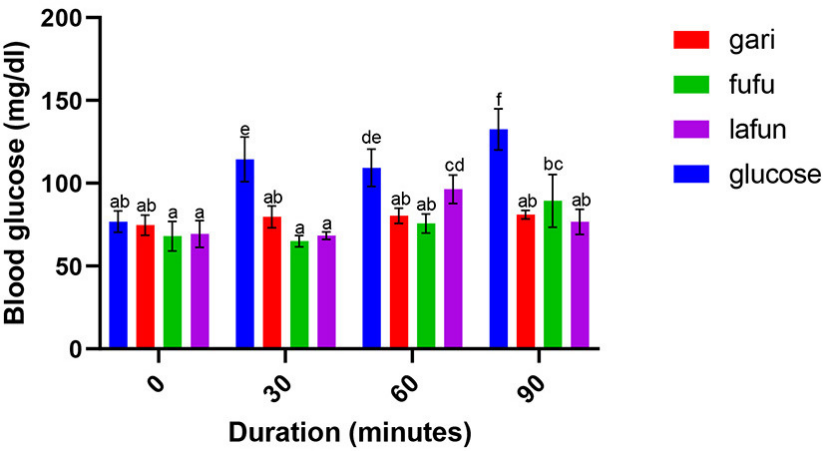
Blood glucose response after the intake of *fufu, lafun and gari* from IITA-TMS-IBA070337. Mean values with different letters are significantly different at *p* < 0.05.

**Figure 3:**
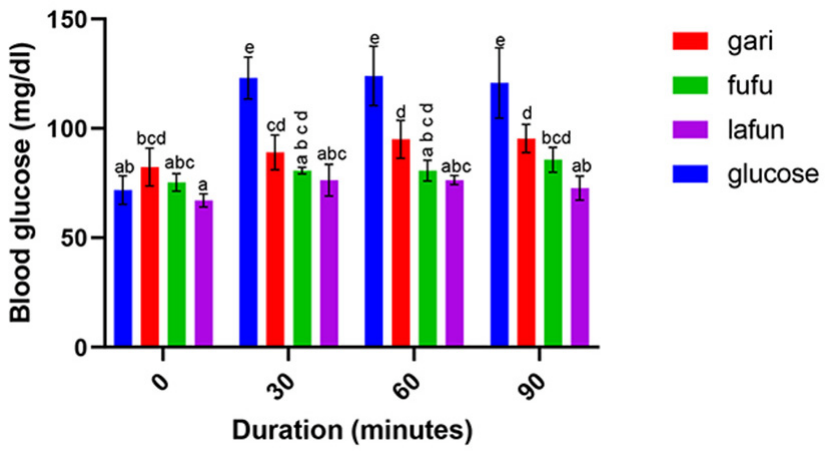
Blood glucose response after the intake of *fufu, lafun and gari* from IITA-TMS-IBA182961. Mean values with different letters are significantly different at *p* < 0.05.

**Figure 4:**
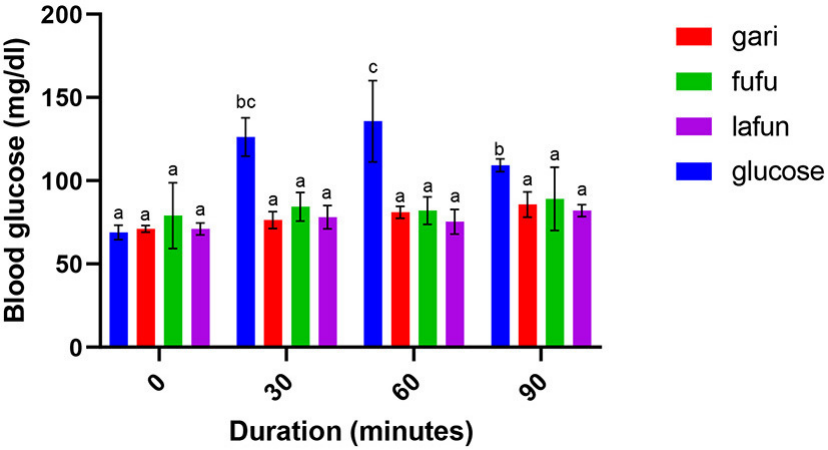
Blood glucose response after the intake of *fufu, lafun and gari* from IITA-TMS-IBA182962. Mean values with different letters are significantly different at *p* < 0.05.

**Figure 5:**
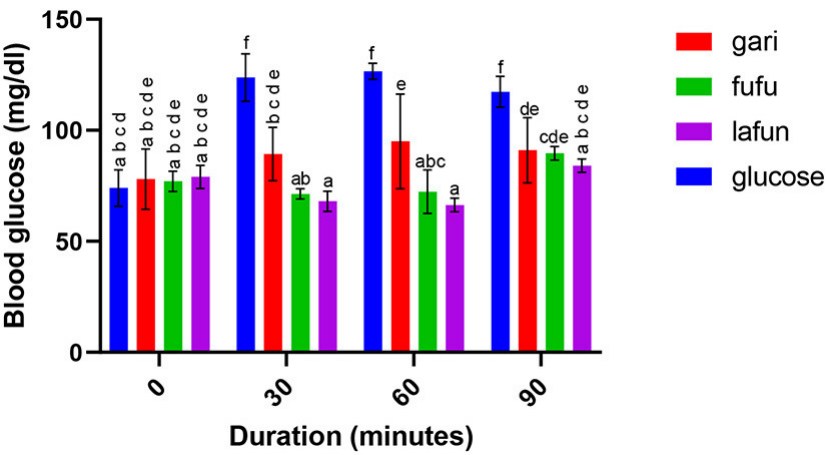
Blood glucose response after the intake of *fufu, lafun and gari* from IITA-TMS-IBA182986. Mean values with different letters are significantly different at *p* < 0.05.

**Figure 6:**
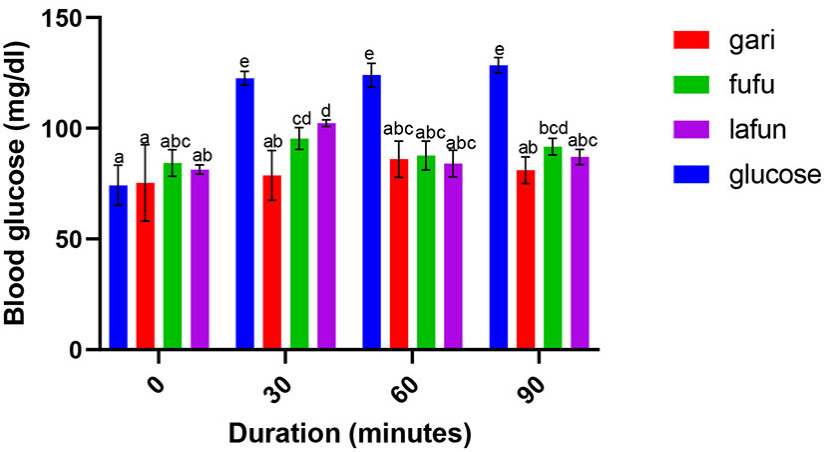
Blood glucose response after the intake of *fufu, lafun and gari* from IITA-TMS-IBA183044. Mean values with different letters are significantly different at *p* < 0.05.

The blood glucose responses resulting from the consumption of the *fufu, lafun* and *gari* from the YFCR genotypes are shown in Figures 2–6. Overall, the *lafun* from different genotypes had the lowest glucose response, increasing the blood glucose by +5 mg/dl at the end of 19 min.

## Discussion

### Bioactive constituents of *fufu, lafun and gari* processed from five YFCR genotypes

The total carotenoid contents of the *fufu, lafun and gari* from the five genotypes of YFCR in this study varied among the products. This agrees with the report of Maziya-Dixon et al. (11), which indicated that the total carotenoids content of yellow-fleshed cassava genotypes was affected by traditional processing methods. The range of total carotenoid concentration (0.79–10.16 μg/g) in *fufu* from the five YFCR genotypes in this study is within the range of total carotenoid concentration (5.82–18.23 μg/g) previously reported for uncooked *fufu* produced from three genotypes (TMS 94/0006, TMS 01/1235 and TMS 01/1371) of yellow-fleshed cassava (11). Similarly, the range of total carotenoid (0.38–9.31 μg/g) obtained in *gari* from the five YFCR genotypes in this study agree with the mean total carotenoid concentration (9.22 μg/g). Maziya-Dixon et al. (11) reported for TMS 94/0006, TMS 01/1235 and TMS 01/1371. However, relative to the total carotenoid concentrations reported by Oliveira et al. (23, 24) for fresh roots of bitter yellow cassava and sweet yellow cassava cultivars (3.65–18.92 and 2.64–14.15 μg/g, respectively) the total carotenoid levels of the traditional products from the five yellow-fleshed cassava genotypes in this study are low, suggesting a possible loss of carotenoids. Different processing methods have been reported to have different impacts on carotenoids composition of processed food (23). Aman et al. (25) reported that food processing could cause losses of the carotenoids composition of food due to degradation, isomerization, and oxidation. Also, processing can disrupt the plant matrix, including the cellular compartments and binding proteins that protect and stabilize the carotenoid pigments. The relatively high carotenoid contents of the different products after processing to various traditional products as revealed in this study support the prospect of yellow-fleshed cassava roots as a viable staple for alleviating vitamin A deficiency and oxidative stress. Carotenoids compounds have been reported to have several health-related properties such as enhancement of immune function, blockade of mammary tumor growth, and protection against blindness caused by age-related macular degeneration (AMD) (26, 27).

As observed in this study, the low total phenolics, tannins, and total flavonoids levels in the cassava products can be attributed to the effect of different processing methods such as boiling, fermentation and roasting on polyphenolics (28). Tannins and flavonoids as phenolic compounds, have been reported to possess antioxidant activities, conferred by their redox properties, which enable them to act as singlet oxygen quenchers, hydrogen donors and reducing agents (29). These compounds also possess digestive enzymes inhibitory activity, due to their high affinity for proteins *via* hydrogen and hydrophobic bonding, enabling them to inhibit enzymes (30, 31). Other health benefits attributed to phenolic compounds include anti-obesity, anti-diabetic and anti-hypertensive, anti-carcinogenic and antimutagenic potentials due to their antioxidative property (32). In particular, different subclasses of flavonoid were reported to structurally exhibit antihyperlipidaemic and antidiabetic activities by mitigating the metabolism of lipid and glucose in metabolic syndrome (33).

### Antioxidant activity of *fufu, lafun and gari* processed from five YFCR genotypes

The ability of *fufu, lafun and gari* to scavenge DPPH^•^ and ABTS^•+^ represent their free radicals-scavenging capacity and ability to serve as antioxidant foods. DPPH^•^ is known as a stable free radical, and its reaction with antioxidant substances convert it to α,α-diphenyl-β-picryl hydrazine. In contrast, ABTS^•+^ are less stable and more reactive than DPPH radicals. Unlike the reactions with DPPH radical, which involves H atom transfer, the reactions with ABTS radicals consist of an electron transfer process (34). Thus, the ability of the *lafun, fufu* and *gari* from the five genotypes of YFCR evaluated in this study to scavenge DPPH^•^ and ABTS^•+^ suggests that they have the potential to prevent the cellular production of free radicals and/or mopping them up in the cell when consumed (35). The reducing power (from Fe^3+^ to Fe^2+^) of the *fufu, lafun and gari* suggests that they may help alleviate the Fe^2+^-mediated generation of hydroxyl radical (OH^•^) from hydrogen peroxide (H_2_O_2_) (36), and Fe^2+^-catalyzed lipid oxidation (37), thereby protecting against oxidative stress.

Among the three products, *lafun* had the strongest antioxidant activity, while *fufu* had the weakest antioxidant activity. The variations in the antioxidant activity of the products from different the YFCR genotypes might be attributed to their different processing methods (28) and genotypic differences (38). The ability of *lafun, fufu* and *gari* to scavenge free radicals and reduce iron (III) (Fe^3+^) to iron (II) (Fe^2+^) might be attributed to bioactive constituents, including carotenoids and polyphenols, which are known to possess antioxidant activity (39).

### Starch-digesting enzymes inhibitory activity of *fufu, lafun* and *gari* processed from five YFCR genotypes

The inhibition of dietary starch-digesting enzymes (α-amylase and α-glucosidase) is a well-established strategy for determining the ability of food products to lower postprandial blood glucose response. It is indicative of the anti-hyperglycaemic activity of the food (31). Alpha-amylase and α-glucosidase are responsible for digesting dietary starch. Alpha-amylase catalyzes the initial step during the starch hydrolysis process. Therefore, the catalytic activity of α-amylase decides the rate of starch digestion to a large extent. On the other hand, alpha-glucosidase hydrolyzes the initially digested products of starch, including maltose, maltotriose, and dextrin, into final absorbable monosaccharides such as glucose and fructose (40).

The α-amylase and α-glucosidase inhibitory ability of the three products differed, with l*afun* having the strongest inhibitory effect on both α-amylase and α-glucosidase. As explained for the antioxidant activity of the products, this variation may be due to the impact of their different processing methods. Reducing the hydrolysis rate of starch through the inhibition of digestive enzymes (α-amylase and α-glucosidase) is an important strategy for relieving postprandial hyperglycaemia (41). Thus, the stronger inhibitory activity of *lafun* over *fufu* and *gari* on α-amylase and α-glucosidase suggests that it may be beneficial for retarding the release of glucose from dietary starch sources and its subsequent absorption into the bloodstream (42).

### Blood glucose response of *fufu, lafun* and *gari* processed from five YFCR genotypes

The functionality of foods in preventing and managing nutrition-related non-communicable diseases such as T2D is increasingly gaining attention. This attention stems from the evidences proving that the consumption of low GI foods over time can improve blood glucose control in people with diabetes and improve insulin sensitivity in glucose intolerance (4). The results from this study revealed that there was a significant variation in the blood glucose responses of the *fufu, lafun* and *gari* investigated in this study. *Lafun* from IITA-TMS-182986 had the least blood glucose response, increasing blood glucose concentration from 79 to 84 mg/dl in 19 min. However, the *fufu* from IITA-TMS-IBA070337 had the highest glucose response, increasing blood glucose concentration from 68 to 89.33 mg/dl in 9 min. Thus, from this study, the order of blood glucose response for the traditional cassava-based products was *fufu* > *gari* > *lafun*. This supports the contribution made by Taiwo (43) that certain carbohydrates are digested rapidly, releasing their glucose into the bloodstream. However, the blood glucose responses of *fufu, lafun* and *gari* were significantly (*p* < 0.05) lower compared to that of the standard glucose. The variation in the glucose response of these different products also agrees with a previous report that different processing methods increased the digestibility of starch in cell walls and plant polysaccharides and conversely reduced their resistance to enzymatic action during digestion (44). Raigond et al. (45) reported that fermentation and heat processes could modify the physical form of carbohydrates through gelatinization and retrogradation of starch, and these modifications can bring about variations in postprandial glucose and insulin response. Kouassi et al. (46) also reported that genotype and different processing methods affected the blood glucose response of root and tuber crops such as cassava.

## Conclusions

The *fufu, lafun and gari* processed from five genotypes of yellow-fleshed cassava roots (YFCR) displayed appreciable antioxidant and starch-digesting enzymes inhibitory activities *in vitro*. Overall, the *lafun* from different genotypes had the strongest antioxidant, α-amylase and α-glucosidase inhibitory activities. The *lafun* from IITA-TMS-182986 also had the least glucose response, while *fufu* from IITA-TMS-IBA070337 had the highest glucose response. Therefore, *lafun* may be a preferred traditional product of YFCR genotypes for dietary intervention, targeting oxidative stress and postprandial hyperglycaemia. Further study is recommended to ascertain the mechanisms and interactions of the various bioactive constituents in *lafun* in mediating the antioxidant and anti-hyperglcaemic activities observed in this study.

## Data availability statement

The raw data supporting the conclusions of this article will be made available by the authors, without undue reservation.

## Ethics statement

The studies involving human participants were reviewed and approved by Kwara State Ministry of Health, Ilorin, Nigeria with Ethical clearance number MOH/KS/EU/777/497. The patients/participants provided their written informed consent to participate in this study.

## Author contributions

EAl and EI: conceptualization. EI, EAl, BK, and MA: methodology. EI and BK: software and writing—original draft preparation. BK and MA: formal analysis, investigation, and data curation. AA, BM-D, EP, EAl, and EAj: resources and writing—review and editing. EI, EAl, and EAj: supervision. EAl and AA: project administration. EAl, AA, EP, and BM-D: fund acquisition. All authors have read and agreed to the published version of the manuscript.

## Funding

This research and the APC were funded by the CGIAR Research Program on Roots, Tubers and Bananas (RTB) and the Bill & Melinda Gates Foundation (BMGF) through OPP1019962.

## Conflict of interest

The authors declare that the research was conducted in the absence of any commercial or financial relationships that could be construed as a potential conflict of interest.

## Publisher's note

All claims expressed in this article are solely those of the authors and do not necessarily represent those of their affiliated organizations, or those of the publisher, the editors and the reviewers. Any product that may be evaluated in this article, or claim that may be made by its manufacturer, is not guaranteed or endorsed by the publisher.

## References

[1] IDF. IDF Diabetes Atlas 2015. 7th Ed. Brussels: International Diabetes Federation (2015). 35914061

[2] Mutyambizi C Pavlova M Chola L Hongoro C Groot W. Cost of diabetes mellitus in Africa: a systematic review of existing literature. Global Health. (2018) 14:3. 10.1186/s12992-017-0318-5 29338746 PMC5771003

[3] Wang S Copeland L. Molecular disassembly of starch granules during gelatinization and its effect on starch digestibility: a review. Food Funct. (2013) 4:1564–80. 10.1039/c3fo60258c 24096569

[4] Thomas DE Elliott EJ. The use of low-glycemic index diets in diabetes control. Brit J Nutr. (2010) 104:797–802. 10.1017/S0007114510001534 20420752

[5] Dalar A Konczak I. Phenolic contents, antioxidant capacities and inhibitory activities against key metabolic syndrome relevant enzymes of herbal teas from Eastern Anatolia. Indust Crop Prod. (2013) 4:383–90. 10.1016/j.indcrop.2012.11.037

[6] Talsma EF Mwangi AM Mburu-de Wagt A Brouwer I Melse A. Yellow cassava and vitamin A status in Kenyan school children: proposed investigation design. Ann Nutr Metab. (2009) 55:378.

[7] Gammone MA Pluchinotta FR Bergante S Tettamanti G D'Orazio N. Prevention of cardiovascular diseases with carotenoids. Front Biosci. (2017) 9:165–71. 10.2741/s480 27814582

[8] Agarwal S Rao AV. Carotenoids and chronic diseases. Drug Metabol Drug Int. (2000) 17:189–210. 10.1515/DMDI.2000.17.1-4.189 11201295

[9] Seifried HE Anderson DE Fisher EI Milner JA. A review of the interaction among dietary antioxidants and reactive oxygen species. J Nutr Biochem. (2007) 18:567–79. 10.1016/j.jnutbio.2006.10.007 17360173

[10] Saltzman A Birol E Bouis HE Boy E De Moura FF Islam Y. Biofortification: progress toward a more nourishing future. Glob Food Secur. (2013) 2:9–17. 10.1016/j.gfs.2012.12.003

[11] Maziya-Dixon B Dixon AGO Ssemakula G. Changes in total carotenoid content at different stages of traditional processing of yellow-fleshed cassava genotypes. Int J Food Sci Technol. (2009) 44:2350–7. 10.1111/j.1365-2621.2007.01638.x

[12] Sanni LO Adebowale AA Filani TA Oyewole OB Westby A. Quality of flash and rotary dried fufu flour. J Food Agricult Environ. (2006) 4:74–8. 10.1234/4.2006.920

[13] Cassbiz. (2018). Available online at: http://www.cassavabiz.org/postharvest (accessed February 1, 2018).

[14] Abass AB Dziedzoave NT Alenkhe BE James BD. Quality Management Manual for the Production Gari. Ibadan: IITA (2013).

[15] Chan EWC Lim YY Chew YL. Antioxidant activity of Camellia sinensis leaves and tea from a lowland plantation in Malaysia. Food Chem. (2007) 102:1214–22. 10.1016/j.foodchem.2006.07.009

[16] Joslyn MA. Tannins NAD related phenolics. In: methods in food analysis 701-725. J Cell Biochem. (1970) 22:188–9.

[17] Asha K Sucheta G Kavita M Nirmala D Jyoti S. Quantification of phenolics and flavonoids by spectrophotometer from-Juglans regia. Int J Pharma Bio Sci. (2010) 1.

[18] Re R Pellegrini N Proteggente A Pannala A Yang M Rice-Evans C. Antioxidant activity applying an improved ABTS radical cation decolorization assay. Free Rad Biol Med. (1999) 26:1231–7. 10.1016/S0891-5849(98)00315-3 10381194

[19] Cervato G Carabelli M Gervasio S Cittera A Cazzola R Cestaro B. Antioxbdant properties of oregano (*Origanum vulgare*) leaf extracts. J Food Biochem. (2000) 24:453–65. 10.1111/j.1745-4514.2000.tb00715.x29729962

[20] Oyaizu M. Studies on products of browning reaction: antioxidative activity of products of browning reaction prepared from glucosamine. Japan J Nutr. (1986) 44:307–15. 10.5264/eiyogakuzashi.44.307

[21] Kwon YI Apostolidis E Shetty K. Inhibitory potential of wine and tea against α-amylase and a-glucosidase for management of hyperglycemia linked to type 2 diabetes. J Food Biochem. (2008) 32:15–31. 10.1111/j.1745-4514.2007.00165.x

[22] Kim YM Jeong YK Wang MH Lee WY Rhee HI. Inhibitory effect of pine extract on α-glucosidase activity and postprandial hyperglycemia. Nutrition. (2005) 21:756–61. 10.1016/j.nut.2004.10.014 15925302

[23] Carvalho LJ Oliveira AG Godoy RO Pacheco S Nutti M de Carvalho JV. Retention of total carotenoid and β-carotene in yellow sweet cassava (Manihot esculenta Crantz) after domestic cooking. Food and Nutr Res. (2012) 56:15788. 10.3402/fnr.v56i0.15788 22468142 PMC3314373

[24] Oliveira RA De Carvalho ML Nutti RM De Carvalho LJ. Assessment and degradation study of total carotenoid and-carotene in bitter yellow cassava (Manihot esculenta Crantz) varieties. Afric J Food Sci. (2010) 4:148–55.

[25] Aman R Schieber A Carle R. Effects of heating and illumination on trans–cis isomerization and degradation of β-carotene and lutein in isolated spinach chloroplasts. J Agric Food Chem. (2005) 53:9512–8. 10.1021/jf050926w 16302770

[26] Chew BP Park JS. Carotenoid action on the immune response. J Nutr. (2004) 134:257S−61S. 10.1093/jn/134.1.257S 14704330

[27] Ahmed SS Lott MN Marcus DM. The macular xanthophylls. Surv Ophthalmol. (2005) 50:183–93. 10.1016/j.survophthal.2004.12.009 15749308

[28] Akinsola OT Alamu EO Otegbayo BO Menkir A Maziya-Dixon B. Nutritional properties of ogi powder and sensory perception of ogi porridge made from synthetic provitamin A maize genotype. Front Nutr. (2021) 8:685004. 10.3389/fnut.2021.685004 34249994 PMC8267175

[29] Chang ST Wu JH Wang SY Kang PL Yang NS Shyur LF. Antioxidant activity of extracts from Acacia confusa bark and heartwood. J Agric Food Chem. (2001) 49:3420–4. 10.1021/jf0100907 11453785

[30] Villiger A Sala F Suter A Butterweck V. *In vitro* inhibitory potential of cynara scolymus, silybum marianum, taraxacum officinale, and peumus boldus on key enzymes relevant to metabolic syndrome. Phytomedicine. (2015) 22:138–44. 10.1016/j.phymed.2014.11.015 25636882

[31] Irondi EA Ajani EO Aliyu OM Olatoye KK Abdulameed HT. Ogbebor OF. Bioactive components, enzymes inhibitory and antioxidant activities of biofortified yellow maize (Zea mays L.) and cowpea (Vigna unguiculata L. Walp) composite biscuits. - The Annals of the University Dunarea de Jos of Galati, Fascicle VI. Food Technol. (2021) 45:86–101. 10.35219/foodtechnology.2021.1.06

[32] Irondi EA Agboola SO Boligon AA. Inhibitory effects of tropical almond leaf extract on xanthine oxidase, pancreatic lipase, and angiotensin 1-converting enzyme, in vitro. J Food Biochem. (2018) 42:e12481. 10.1111/jfbc.12481

[33] Zhang J Zhao L Cheng Q Ji B Yang M Sanidad KZ. Structurally different flavonoid subclasses attenuate high-fat and high-fructose diet induced metabolic syndrome in rats. J Agricul Food Chem. (2018) 66:12412–20. 10.1021/acs.jafc.8b03574 30360615

[34] Srikanth G Babu SM Kavitha CHN Rao MB Vijaykumar N Pradeep CH. Studies on in-vitro antioxidant activities of Carica papaya aqueous leaf extract. Res J Pharm Biol Chem Sci. (2010) 1:59–65.

[35] Irondi EA Adebara OO Olateju A Boligon AA. Phenolic constituents, anti-radicals, and enzymes inhibitory potentials of Brachystegia eurycoma seeds: effects of processing methods. Int J Food Prop. (2018) 20:S3004–14. 10.1080/10942912.2017.1396340

[36] Oboh G Raddatz H Henle T. Antioxidant properties of polar and non-polar extracts of some tropical green leafy vegetables. J Sci Food Agric. (2008) 88:2486–92. 10.1002/jsfa.3367

[37] Hsu CL Chen W Weng YM Tseng CY. Chemical composition, physical properties, and antioxidant activities of yam flours as affected by different drying methods. Food Chem. (2003) 83:85–92. 10.1016/S0308-8146(03)00053-0

[38] Alamu EO Maziya-Dixon B Menkir A Irondi EA Olaofe O. Bioactive composition and free radical scavenging activity of fresh orange maize hybrids: impacts of genotype, maturity stages, processing methods. Front Nutr. (2021) 8:640563. 10.3389/fnut.2021.640563 33718422 PMC7943467

[39] Elemosho AO Irondi EA Alamu EO Ajani EO Menkir A Maziya-Dixon B. Antioxidant and starch-hydrolyzing enzymes inhibitory properties of striga-resistant yellow-orange maize hybrids. Molecules. (2021) 26:6874. 10.3390/molecules26226874 34833966 PMC8617639

[40] Irondi EA Ogunsanmi AO Ahmad RS Ajani EO Adegoke BM Boligon AA. Effect of roasting on phenolics composition, enzymes inhibitory and antioxidant properties of cowpea pulses. J Food Meas Charact. (2019) 13:1489–96. 10.1007/s11694-019-00064-0

[41] Yilmazer-Musa M Griffith AM Michels AJ Schneider E Frei B. Grape seed and tea extracts and catechin 3-gallates are potent inhibitors of a-amylase and a-glucosidase activity. J Agric Food Chem. (2012) 60:8924–9. 10.1021/jf301147n 22697360 PMC4356113

[42] Tucci SA Boyland EJ Halford JC. The role of lipid and carbohydrate digestive enzyme inhibitors in the management of obesity: a review of current and emerging therapeutic agents. Diabetes Metab Synd Obesity Targets Therapy. (2010) 3:125. 10.2147/DMSO.S7005 21437083 PMC3047983

[43] Taiwo KA. Utilization potentials of cassava in Nigeria: the domestic and industrial products. Food Rev. Int. (2006) 22:29–42. 10.1080/87559120500379787

[44] Englyst KN Englyst HN. Carbohydrate bioavailability. Br J Nutr. (2005) 94:1–11. 10.1079/bjn20051457 16115326

[45] Raigond P Ezekiel R Raigond B. Resistant starch in food: a review. J Sci Food Agric. (2015) 95:1968–78. 10.1002/jsfa.6966 25331334

[46] Kouassi NK Tiahou GG Abodo JRF Camara-Cisse M Amani GN. Influence of the variety and cooking method on glycemic index of Yam. Pakis J Nutr. (2009) 8:993–9. 10.3923/pjn.2009.993.999

